# A Physics-Informed Neural Network (PINN) Approach to Over-Equilibrium Dynamics in Conservatively Perturbed Linear Equilibrium Systems

**DOI:** 10.3390/e28010009

**Published:** 2025-12-20

**Authors:** Abhishek Dutta, Bitan Mukherjee, Sk Aftab Hosen, Meltem Turan, Denis Constales, Gregory Yablonsky

**Affiliations:** 1 Department of Chemical Engineering, Izmir Institute of Technology, Izmir 35430, Turkey; 2 Department of Chemical Engineering, Jadavpur University, Kolkata 700032, India; 3 Department of Mathematics, Ege University, Izmir 35180, Turkey; 4 Department of Electronics and Information Systems, Ghent University, Building S-8, Krijgslaan 281, B-9000 Ghent, Belgium; 5 McKelvey School of Engineering, Department of Energy, Environmental and Chemical Engineering, Washington University in Saint Louis, St. Louis, MO 63130, USA

**Keywords:** conservatively perturbed equilibrium, cyclic and acyclic mechanisms, over-equilibrium dynamics, physics-informed neural network, finite-time thermodynamics

## Abstract

Conservatively perturbed equilibrium (CPE) experiments yield transient concentration extrema that surpass steady-state equilibrium values. A physics-informed neural network (PINN) framework is introduced to simulate these over-equilibrium dynamics in linear chemical reaction networks without reliance on extensive time-series data. The PINN incorporates the reaction kinetics, stoichiometric invariants, and equilibrium constraints directly into its loss function, ensuring that the learned solution strictly satisfies physical conservation laws. Applied to three- and four-species reversible mechanisms (both acyclic and cyclic), the PINN surrogate matches conventional ODE integration results, reproducing the characteristic early concentration extrema (maxima or minima) in unperturbed species and the subsequent relaxation to equilibrium. It captures the timing and magnitude of these extrema with high accuracy while inherently preserving total mass. Through the physics-informed approach, the model achieves accurate results with minimal data and a compact network architecture, highlighting its parameter efficiency.

## 1. Introduction

Conservatively perturbed equilibrium (CPE) is a kinetic phenomenon that uses the stability of chemical equilibrium at a fixed temperature, in which a reacting mixture is initiated from a specially constructed state that respects all elemental balances but reaches one (or more) species exactly in its equilibrium concentration [1]. The remaining species are “conservatively” displaced from equilibrium while preserving the totals of each chemical element obeying elemental conservation. The ensuing relaxation to equilibrium exhibits an unavoidable, well-defined extremum (maximum or minimum) in the concentration of every “unperturbed” species before the system finally settles at the unique, stable equilibrium composition. This phenomenon and its core properties were initially formulated for general mechanisms in a closed system and then developed for reactor models, providing a rigorous and robust framework that differs fundamentally from small-signal relaxation methods [2] because the perturbations are finite. Operationally, CPE proceeds in five steps: (1) compute the equilibrium composition at the chosen T (and p, if relevant); (2) select at least two species to perturb away from their equilibrium values; (3) select at least one “unperturbed” species whose initial concentration equals its equilibrium value; (4) enforce all elemental balances so that the total number of atoms remain unchanged; and (5) integrate the kinetic model and track the trajectories until equilibrium is reached. These steps apply in batch systems and can be carried over to flow reactors, provided the conservative balances are respected in the inlet mixture.

A linear reversible mechanism *A* ⇌ *B* ⇌ C has a unique equilibrium composition subject to the conservation of total concentration. If the system is initially at equilibrium with respect to the intermediate species *B*, and the terminal species *A* and *C* are perturbed while respecting the conservation constraint, then the time evolution of B(t) exhibits an extremum. A key property of this extremum is that the time at which it occurs depends only on the kinetic rate constants and not on the magnitude or direction of the perturbation. This invariance is the defining feature of CPE. For nonlinear reversible mechanisms (e.g., when mass-action kinetics involve nonlinearities such as bimolecular association, enzyme saturation, or autocatalysis), CPE also applies, but with modifications. If a system at equilibrium is perturbed within its conservation constraints, certain intermediate species can exhibit extrema in their relaxation profiles. However, unlike the linear case, the time at which the extremum occurs generally depends on both the rate constants and the equilibrium composition. The underlying reason is that the Jacobian of the nonlinear system at equilibrium governs the local relaxation dynamics: close to equilibrium the system behaves linearly, so a perturbation-independent extremum emerges in the linear response regime, but nonlinearities introduce amplitude dependence as the perturbation grows larger. CPE extends beyond simple sequences to general cyclic and acyclic networks and, more importantly, to flow reactors such as CSTR and PFR. In this case, the extremum that the unperturbed species experiences can lie above its thermodynamic equilibrium value, i.e., an “over-equilibrium” whose magnitude is tunable by the size of the initial perturbations while still maintaining conservation laws [3,4]. Two mechanistic inferences follow from this extremum. First, if an unperturbed species participates in only one step, the concentration extremum coincides with a momentary (instantaneous) equilibrium of that step; if it participates in multiple steps, the extremum generally does not represent a momentary equilibrium for any single step. Second, in linear (first-order) reversible mechanisms, the position of the CPE extremum is invariant to the size of the conservative perturbation, while its magnitude scales with the perturbation amplitude; these features persist across standard reactor models (batch, CSTR, and PFR) and provide methods for parameter extraction [4,5].

Physics-informed neural networks (PINNs) are a class of function approximators that embed governing equations, typically ordinary or partial differential equations, into the learning objective so that the neural network’s predictions satisfy data and physics simultaneously. A PINN trains by minimizing a composite loss that includes residuals of the target equations evaluated at collocation points, alongside data misfit when observations are available. In their foundational study, Raissi et al. [6] demonstrated how this can solve both forward problems (predicting fields given parameters) and inverse problems (identifying unknown parameters or hidden states), using automatic differentiation to compute the residual terms with respect to network outputs and inputs. Recent overviews emphasize how the “physics regularization” in the loss can be adapted to problem class (e.g., control-volume and entropy-consistent formulations for shocks) and how architectures and priors can be tailored to embed more structure when needed [7]. This structure naturally supports inverse modeling for kinetics: by differentiating the residual with respect to θ during gradient-based training, the network can estimate Arrhenius parameters or effectiveness factors while reconstructing species trajectories. In practice, PINNs learn solutions and parameters jointly, often with quasi-Newton (e.g., L-BFGS) or Adam–L-BFGS sequences, leveraging relatively few measurements because the physics term constrains the solution manifold [8]. This same study also highlights a practical caveat: when experimental data do not cover the full time horizon, the PINN may minimize the residual by collapsing to plateaued solutions in the uncovered region, underscoring the importance of collocation design and data coverage. Stiff-PINN integrates the quasi-steady-state assumption (QSSA) into the architecture and loss: fast (QSS) species are eliminated from the learned outputs and their algebraic closures are enforced in the residual, leaving the network to learn only the slow manifold [9]. Simılarly, the Multiscale PINN (MPINN) also aims to restructure the learning task rather than the physics: it segregates species by time scale, fits each group with a dedicated subnetwork, and uses adaptive loss reweighting keyed to species-specific residual indicators [10]. Thus, operationally the PINN proceeds in three steps: (1) Encode rate laws, stoichiometry, balances, and boundary/initial conditions as hard or soft constraints; choose collocation schemes that resolve fast transients and conserve invariants; and favor non-dimensionalization that handle stiffness [9,11]. (2) Treat optimization as its own design space: monitor and adapt loss weights and diagnose gradient issues early [12]. (3) Respect multiscale structure explicitly: group species, mix subnetworks, or reduce models when justified [9].

Recent studies [13,14,15] show that physics-informed neural networks (PINNs) are becoming a versatile tool across chemical engineering, complementing classical first-principles models in both steady-state and dynamic applications. Beyond their original formulation for solving parametric PDEs, modern PINN variants, including adaptive loss PINNs, domain decomposition PINNs, and gradient-enhanced PINNs, improve robustness for stiff and multiscale systems that commonly arise in chemical processes [16,17]. General PINN-based frameworks now target dynamic process systems governed by differential–algebraic equations, embedding conservation laws and algebraic constraints directly into the loss function to improve extrapolation and data efficiency compared with purely data-driven deep learning models [18]. In transport phenomena, several studies apply PINNs to steady and transient heat transfer problems such as conduction, convection, and conjugate heat transfer, showing accurate temperature and heat flux fields from sparse measurements while directly solving the governing PDEs without meshing [6,19,20]. In reactor engineering, forward and inverse PINNs have been developed for isothermal fixed-bed CO_2_ methanation reactors, enabling simultaneous solution of the reactor model and parameter identification under realistic operating conditions [21]. Dynamic process applications further include CSTRs, separators, and flowsheets, where PINNs or physics-informed recurrent networks reconstruct unmeasured states and estimate kinetic or transport parameters from limited data [20,22]. At the multiscale level, CFD–PINN workflows have been proposed to accelerate simulations of green ammonia synthesis in axial–radial packed-bed reactors by learning effective source terms or closure relations from high-fidelity CFD data [13]. Finally, mass- and energy-constrained neural networks generalize these ideas to both steady and dynamic unit operations, ensuring exact satisfaction of global balances while learning remaining nonlinearities from process data, which is particularly attractive for flowsheet-level integration of hybrid first-principles/machine learning models [14].

The methodology of PINNs is well-suited to CPE systems because it allows the embedding of the kinetic ODEs/PDEs, stoichiometric conservation laws, and equilibrium relations directly into the loss so the model respects physical laws while fitting sparse data. For CPE specifically, the equilibrium relations and the conservative constraints can be enforced as hard constraints (via parameterization) or soft penalties, enabling the model to learn over-equilibrium deviations and their routes without violating mass or charge balances. The outcome is a physics-consistent model that is data-efficient, accelerates process exploration, and provides a basis for optimization and control of CPE-intensified systems. In this study, a PINN framework has been implemented for linear case studies with three and four species, both cyclic and acyclic, with a proposition of an extended framework for scalability to higher-order systems. The specific contributions of this study are threefold: (1) Application of PINNs to CPE systems, demonstrating that physics-informed learning can capture over-equilibrium dynamics and concentration extrema without dense time-series data; (2) physics-consistent loss function design that enforces stoichiometric invariants, equilibrium constraints, and kinetic ODEs simultaneously, ensuring mass conservation and thermodynamic consistency; and (3) systematic validation across linear mechanisms of increasing complexity (three- and four-species, acyclic and cyclic), establishing parameter efficiency and accuracy benchmarks for extending PINNs to finite-time thermodynamic phenomena.

## 2. Methodology

### 2.1. Conservatively Perturbed Equilibrium (CPE)

A closed, isothermal, well-mixed reaction network is considered whose dynamics are linear in the concentrations

(1) $$\frac{dc(t)}{dt}=Mc\left(t\right),\;c\left(t\right)\in R^{N},$$

where *M* is the kinetic generator assembled from elementary forward and reverse rate constants. The equilibrium composition $c_{eq}$ satisfies $Mc_{eq}=0$. Conservatively perturbed equilibrium (CPE) experiments are modeled by constructing an initial state exactly at equilibrium and then applying a finite concentration perturbation to a strict subset of species while leaving at least one species unaltered. Let P⊂{1,…,N} denote indices of perturbed species and U its complement (unperturbed). The CPE initial condition is

(2) $$c\left(0\right)=c_{eq}+\Delta ,\;\Delta_{i}=0\;\left(i\in U\right),\;\Delta \in C,$$

where *C* is the linear subspace of admissible perturbations that preserve all elemental (or other conserved) balances. Writing those invariants as rows of a matrix *L*, admissibility reads LΔ=0. In the present linear batch setting, the system returns to the same $c_{eq}$; the transient encodes the mechanism through the interplay of the eigenstructure of *M* and the choice of Δ. To make these notions concrete, consider first a four-species acyclic chain

(3) $$A\underset{k_{1}^{-}}{\overset{k_{1}^{+}}{⇌}}B\underset{k_{2}^{-}}{\overset{k_{2}^{+}}{⇌}}C\underset{k_{3}^{-}}{\overset{k_{3}^{+}}{⇌}}D,$$

with concentrations $c={\left[A,B,C,D\right]}^{T}$. Using the mass-action rates $r_{1}=k_{1}^{+}A-k_{1}^{-}B$, $r_{2}=k_{2}^{+}B-k_{2}^{-}C$, $r_{3}=k_{3}^{+}C-k_{3}^{-}D$, the dynamics are $\dot{A}=-r_{1}$, $\dot{B}=r_{1}-r_{2}$, $\dot{C}=r_{2}-r_{3}$, and $\dot{D}=r_{3}.$ Equivalently,

(4) $$\dot{c}=Mc,\;M=\left[\begin{array}{cccc} -k_{1}^{+} & k_{1}^{-} & 0 & 0\\ k_{1}^{+} & -(k_{1}^{-}+k_{2}^{+}) & k_{2}^{-} & 0\\ 0 & k_{2}^{+} & -(k_{2}^{-}+k_{3}^{+}) & k_{3}^{-}\\ 0 & 0 & k_{3}^{+} & -k_{3}^{-} \end{array}\right].$$

The equilibrium vector $c_{eq}$ is the normalized right null vector of *M*. Because the network is closed, one may work with normalized concentrations so that $1^{T}c\left(t\right)=1$ for all t; i.e.,

(5) $$1^{T}c\left(t\right)=\sum_{i=1}^{4}c_{i}\left(t\right)=1,\;1={\left[1,1,1,1\right]}^{T}.$$

In that normalization the admissible perturbations satisfy $1^{T}\Delta =0$ in addition to any elemental balances encoded in *L*. A CPE realization on this chain perturbs, for example, the end species *A* and *D* while leaving *B* and *C* strictly at their equilibrium values: $\Delta_{B}=\Delta_{C}=0$. Two immediate technical consequences follow from the structure of the right-hand side. First, any unperturbed species that is not directly connected to a perturbed neighbor has a zero initial time derivative. Indeed, evaluating $\dot{B}\left(0\right)=r_{1}\left(0\right)-r_{2}\left(0\right)$ with $B\left(0\right)=B_{eq}$, $C\left(0\right)=C_{eq}$, $A\left(0\right)=A_{eq}+\Delta_{A}$, and $D\left(0\right)=D_{eq}+\Delta_{D}$, one has

(6) $$\begin{array}{cc} r_{1}\left(0\right) & =k_{1}^{+}\left(A_{eq}+\Delta_{A}\right)-k_{1}^{-}B_{eq}=\underset{=0}{\underbrace{k_{1}^{+}A_{eq}-k_{1}^{-}B_{eq}}}+k_{1}^{+}\Delta_{A}=k_{1}^{+}\Delta_{A},\;and\\ r_{2}\left(0\right) & =k_{2}^{+}B_{eq}-k_{2}^{-}C_{eq}=0 \end{array}$$

with $\dot{B}\left(0\right)=k_{1}^{+}\Delta_{A}$. If $\Delta_{A}=0$ as well (i.e., neither neighbor of B is perturbed), then $\dot{B}\left(0\right)=0$. The same reasoning applies to *C*. This “initial shielding” is a sharp, local diagnostic at t=0: it depends only on which species were perturbed and on network connectivity, not on the perturbation magnitude. Second, whenever an end species (such as *A* or *D*) is held unperturbed at t=0, any turning point of its trajectory occurs at a momentary balance of the adjacent step. For instance, at an extremum of A(t) one has $\dot{A}=0\Rightarrow r_{1}=0\Rightarrow k_{1}^{+}A=k_{1}^{-}B$. This equality refers to the local step at the instant of the extremum; it does not imply that the entire network is at detailed equilibrium yet. Taken together, these two properties, i.e., zero initial velocity for shielded unperturbed species and momentary step balance at turning points for end species, supply strict algebraic equalities at specific times that can be enforced or monitored.

The same CPE construction can be written for a three-species acyclic chain A⇌B⇌C by removing the third step and the species *D*. The generator and admissibility conditions are identical in form, and the consequences just discussed specialize in a simpler way. If *C* is left unperturbed while *A* and *B* are perturbed in a balance-preserving manner, then $\dot{C}\left(0\right)=k_{2}^{+}B\left(0\right)-k_{2}^{-}C\left(0\right)=k_{2}^{+}\Delta_{B}$; thus $\dot{C}\left(0\right)$ is nonzero precisely when its neighbor is perturbed. If instead *B* is the unperturbed species and only *A* and *C* are perturbed, then $\dot{B}\left(0\right)=k_{1}^{+}\Delta_{A}+k_{2}^{-}\Delta_{C}$, showing how the initial drift of the unperturbed middle species is controlled by the two adjacent perturbations. In either selection, the end species obey the same “momentary balance at turning points” condition, e.g., $\dot{A}=0\Rightarrow k_{1}^{+}A=k_{1}^{-}B$. For a three-species cyclic network,

(7) $$A\underset{k_{1}^{-}}{\overset{k_{1}^{+}}{⇌}}B\underset{k_{2}^{-}}{\overset{k_{2}^{+}}{⇌}}C\underset{k_{3}^{-}}{\overset{k_{3}^{+}}{⇌}}A,$$

the generator *M* becomes circulant-like, with nonzeros wrapping from *C* to *A*. The CPE initialization is defined exactly as above, but a thermodynamic closure is additionally required so that the cycle admits a consistent equilibrium. Writing equilibrium constants $K_{i}=k_{i}^{+}/k_{i}^{-}$, the standard loop constraint

(8) $$K_{1}K_{2}K_{3}=1\;\left(\mathrm{equivalently}\;\sum \Delta G_{i}^{\circ }=0\right)$$

ensures that a positive null vector $c_{eq}$ exists and is unique up to normalization. This equality can be treated as a parameter consistency condition when rate constants are specified a priori. Because each species now participates in two steps, the “end species momentary balance” observation no longer applies; nevertheless, the initial shielding logic still holds: an unperturbed species whose two neighbors are both unperturbed has ${\dot{c}}_{i}\left(0\right)=0$, while perturbing at least one neighbor introduces a nonzero initial drift via the corresponding adjacent rate.

Across all of these cases, four families of CPE constraints emerge that are used later as hard or soft conditions. First, the dynamic law itself must hold at every time, $\dot{c}\left(t\right)-Mc\left(t\right)=0$, which is the formal statement of compliance with the chemical mechanism. Second, the equilibrium anchor must be respected, $c\left(t\right)\to c_{eq}$ as t→∞ with $Mc_{eq}=0$, and any constraints linking the rate constants on cycles (e.g., $K_{1}K_{2}K_{3}=1$) must be satisfied. Third, the conservation relations must hold for all *t*, $Lc\left(t\right)=Lc\left(0\right)=Lc_{eq}$, including, in normalized units, $1^{T}c\left(t\right)=1$. Fourth, the CPE admissibility pattern must be honored at t=0: the chosen unperturbed indices *U* start exactly at equilibrium ($\Delta_{i}=0$ for i∈U), at least two species are perturbed to encode a genuine finite displacement, and LΔ=0 is satisfied.

### 2.2. Physics-Informed Neural Network (PINN)

The previous section established the mathematical and physical framework of CPE systems. Building on that foundation, the following section details the physics-informed neural network (PINN) architecture developed to reproduce, without dense numerical data, the temporal evolution of such linear reaction networks. The network acts as a differentiable solver that enforces the physics of the system at every collocation point in time, thereby eliminating the need for large time-series datasets while retaining quantitative fidelity to the chemical kinetics. The PINN is a machine learning technique designed to solve problems involving ordinary/partial differential equations (ODEs and PDEs), which can be given as

(9) $$F\left(x,u,\frac{du}{dx},\frac{d^{2}u}{dx^{2}},\frac{\partial u}{\partial x},\frac{\partial^{2}u}{\partial x^{2}},\ldots ,\frac{d^{m}u}{dx^{m}},\frac{du}{dt}\frac{\partial^{m}u}{\partial x^{m}},\frac{\partial u}{\partial t}\right)=0.$$

Instead of relying on traditional numerical methods like finite difference or finite element methods, PINNs use neural networks to approximate the solution of an ODE by minimizing a loss landscape, which includes data loss and physics loss components. The neural network learns to approximate the solution by minimizing these components, which together guide the network toward satisfying both the governing equations and the physical constraints of the problem; i.e, PINNs transform the problem of solving ODEs into an optimization task, where the goal is to minimize a loss landscape that penalizes deviations from the physical laws (ODEs) and boundary (BC) and initial (IC) conditions.

**Figure 1:**
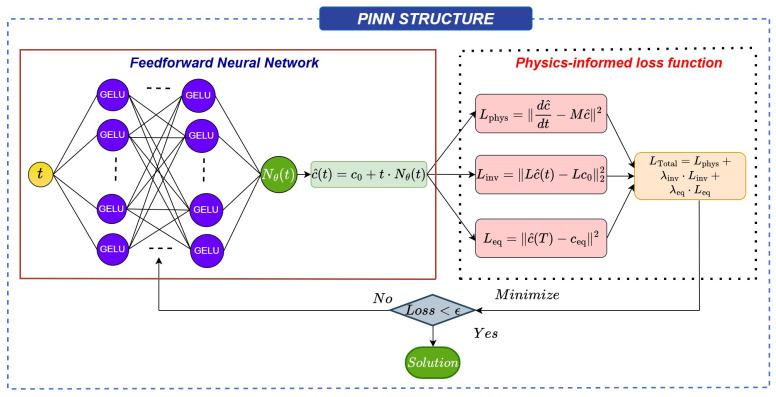
Schematic of physics-informed neural network (PINN) framework for solving a linear case CPE system.

Figure 1 represents the PINN framework used in this study. The inputs of the PINN are the independent variable *x* and time *t*. The output of the PINN is the predicted dependent variable. An artificial neural network (ANN) in PINN $\hat{u}\left(x,t;\theta \right)$ is initially designed as a surrogate for the solution u(x,t). The parameter θ encompasses all the hyperparameters, including the weight matrices W and bias vectors b within the ANN such that

(10) $$\hat{u}\left(x,t;\theta \right)=\sum_{i=1}^{n}W_{i}\cdot f_{i-1}\left(x,t\right)+b_{i},$$

where $f_{i-1}\left(x,t\right)$ is the output from the previous layer, with (x,t) being the input to the network.

Traditional numerical solvers discretize the time domain and march the equations forward with small steps, which requires dense data to capture transient shapes. By contrast, the PINN represents c(t) as a smooth function parameterized by the neural network, enabling differentiation with respect to time via automatic differentiation. This continuous, differentiable representation makes it possible to compute the residuals $\dot{c}\left(t\right)-Mc\left(t\right)$ exactly at arbitrary points in time, enforcing the chemical dynamics throughout the domain without an explicit grid. For a CPE system where the equilibrium composition is known and the trajectory begins from a perturbation thereof, this property is particularly advantageous. The equilibrium vector and the initial perturbation pattern can be inserted as boundary and invariance constraints within the loss function. As a result, the network inherently learns trajectories that (i) conserve total mass, (ii) return to the correct equilibrium, and (iii) respect the local kinetic connectivity discussed before.

Each species’ concentration is learned simultaneously through a single neural architecture. For the systems studied here, the network takes time t∈[0,T] as input and outputs the four-component concentration vector $\hat{c}\left(t\right)=\left[\hat{A},\hat{B},\hat{C},\hat{D}\right]$. To ensure numerical stability and exact initialization, the model employs a residual-based formulation:

(11) $$\hat{c}\left(t\right)=c_{0}+tN_{\theta }\left(t\right),$$

where $c_{0}$ is the known initial concentration vector obtained from the CPE perturbation and $N_{\theta }\left(t\right)$ is a fully connected neural subnetwork with trainable parameters θ. This construction automatically satisfies the initial condition at t=0 ($\hat{c}\left(0\right)=c_{0}$) and prevents spurious offsets. Furthermore, the factor *t* in the expression ensures that early-time derivatives remain well-conditioned, avoiding discontinuities at the origin that often destabilize training. The subnetwork $N_{\theta }\left(t\right)$ consists of *L* hidden layers with H neurons per layer:

(12) t→Linear(1,H)→GELU…Linear(H,H)→GELU→Linear(H,4).

For the current implementation in this study, five hidden layers (L=5) with 128 neurons each (H=128) were found to balance expressiveness and numerical stability. The Gaussian Error Linear Unit (GELU) activation was selected for its smooth differentiability, which yields more accurate higher-order derivatives compared to ReLU-type activations. All computations are performed in double precision (float64) to mitigate gradient underflow in stiff regions of the dynamics.

The central element of the PINN is the physics-informed loss function, which integrates the CPE constraints derived before. For a randomly sampled set of collocation points ${\left\{t_{i}\right\}}_{i=1}^{N_{f}}$ within the time domain, the total loss is defined as follows:

(13) $$L=Lphys+\lambda invLinv+\lambda eqL_{eq},$$

where each component enforces a specific physical requirement. The physics residual loss is defined as follows:

(14) $$L_{phys}=\frac{1}{N_{f}}\sum_{i}{∥\frac{d\hat{c}\left(t_{i}\right)}{dt}-M\hat{c}\left(t_{i}\right)∥}^{2}$$

which ensures that the learned function satisfies the governing kinetic equations at all collocation points. The time derivative $d\hat{c}/dt$ is obtained analytically via automatic differentiation of the neural output with respect to *t*. The invariant loss is defined as follows:

(15) $$L_{inv}=\frac{1}{N_{f}}\sum_{i}{\left(1^{T}\hat{c}\left(t_{i}\right)-1\right)}^{2}$$

which enforces the global conservation of total concentration (or any other balance expressed through the operator $L\hat{c}=Lc_{0}$). Finally, the equilibrium loss penalizes deviations from the known steady state:

(16) $$Leq=∥\hat{c}{\left(T\right)-ceq∥}^{2},$$

with the option to include local equilibrium equalities (e.g., $k_{1}^{+}\hat{A}=k_{1}^{-}\hat{B}$ at turning points) as additional regularization terms. The scalar weights $\lambda_{inv}$ and $\lambda_{eq}$ modulate the relative influence of each contribution; empirical tuning showed that values near 0.5–1.0 provided stable convergence without biasing toward any single term.

#### Training Strategy and Optimization

In this study, training proceeds by sampling $N_{f}=2000$ collocation points uniformly in the time interval [0,T]. Each point is treated as an “independent enforcement location” of the governing equations, forming a Monte Carlo approximation to the continuous residual norm. The optimizer is the Adam stochastic gradient method with an initial learning rate of $10^{-3}$. To improve convergence stability and prevent overshooting near equilibrium, an exponential learning rate scheduler with decay factor γ=0.99 is applied every 1000 epochs. In total, 2000 epochs are run for each system, which is sufficient for the residuals to reach the 10^−5^–10^−6^ range.

The network is implemented in PyTorch (version 2.7.0) using automatic differentiation on double-precision tensors. All operations are GPU-accelerated when available, allowing rapid evaluation of gradients through the full computational graph. To further stabilize training, gradient clipping and zero-bias initialization of the output layer are available, but the reported runs use the default PyTorch initialization; the residual-stable envelope $\left(\hat{c}\left(t\right)=c_{0}+tN_{\theta }\left(t\right)\right)$ already enforces $\left(\hat{c}\left(0\right)=c_{0}\right)$ exactly, keeping initial predicted rates well behaved near t=0. Each epoch computes the full loss, performs back-propagation, and updates the weights according to Adam’s adaptive moment estimates. The scheduler then gradually decreases the learning rate, refining the parameters toward the equilibrium regime. A distinct advantage of this approach is that, because the derivative operator is embedded analytically, the trained model implicitly satisfies smoothness and differentiability requirements, enabling direct evaluation of higher-order quantities such as $\ddot{c}\left(t\right)$ without numerical differencing.

## 3. CPE-PINN Integration

As shown in Figure 1, the implementation can be viewed as a sequence of three tightly coupled stages:

1. Physical initialization: The equilibrium composition $c_{eq}$ is computed from the kinetic matrix *M* as its normalized null vector. A valid CPE perturbation Δ satisfying LΔ=0 is applied to construct the initial state $c_{0}=c_{eq}+\Delta$.
2. Neural approximation and differentiation: The neural architecture $\hat{c}\left(t\right)=c_{0}+tN_{\theta }\left(t\right)$ generates continuous predictions for any *t*; automatic differentiation supplies the exact time derivatives $\dot{\hat{c}}\left(t\right)$.
3. Physics-consistent optimization: The total loss L combines differential residuals, invariance preservation, and equilibrium anchoring. Minimizing L drives the network toward full compliance with both the kinetic law and the CPE constraints.

Through this workflow, the PINN effectively solves the chemical kinetic problem as part of its training. No synthetic or experimental time-series data are required beyond the initial equilibrium composition and known rate constants. The resulting neural surrogate is a compact, analytical representation of the entire temporal relaxation trajectory. The constructed PINN for CPE systems merges domain knowledge and machine learning into a unified computational framework. By encoding the kinetic generator M, the equilibrium vector $c_{eq}$, and the conservation structure directly within its architecture and loss function, the network enforces physical realism at every stage of training. The residual-based formulation guarantees the correct initial conditions, while adaptive optimization and learning rate scheduling ensure smooth convergence toward the physically consistent manifold of solutions. Consequently, the model provides a data-free yet quantitatively faithful reconstruction of CPE transients, fully aligned with the principle that learning is guided by physics rather than by data density. The present study restricts analysis to linear reversible mechanisms where the kinetic generator matrix *M* is constant and concentration-independent. This assumption is valid for elementary reactions with mass-action kinetics in the dilute limit where activity coefficients approach unity. Extension to nonlinear systems would require several modifications: (1) the PINN residual loss would remain valid, as automatic differentiation can compute residuals exactly; (2) the perturbation invariance property of CPE extrema would generally fail, as the system Jacobian depends on initial composition; and (3) the thermodynamic closure constraint $k_{1}k_{2}k_{3}=1$ would be replaced by more complex equilibrium relations.

## 4. Results and Discussion

Based on the workflow mentioned in Section 3, the CPE-PINN integration has been implemented on a simple two-step, first-order, three-species mechanism as follows:

$$A\underset{k_{1}^{-}}{\overset{k_{1}^{+}}{⇌}}B\underset{k_{2}^{-}}{\overset{k_{2}^{+}}{⇌}}C.$$

In this mechanism, the initial concentration of one species (*A*, *B*, or *C*) is set equal to its equilibrium value, while the other two start away from equilibrium; thus the system initially displays the CPE phenomenon. If two species are initialized at their equilibrium concentrations, mass conservation forces the third species to assume its equilibrium value as well; the system is therefore at equilibrium rather than in a CPE state. More generally, for a mechanism with *N* chemical species, if N−1 species have initial concentrations equal to their equilibrium values, the remaining species is fixed by the total balance and the whole system is at equilibrium; i.e., the CPE phenomenon cannot arise.

**Figure 2:**
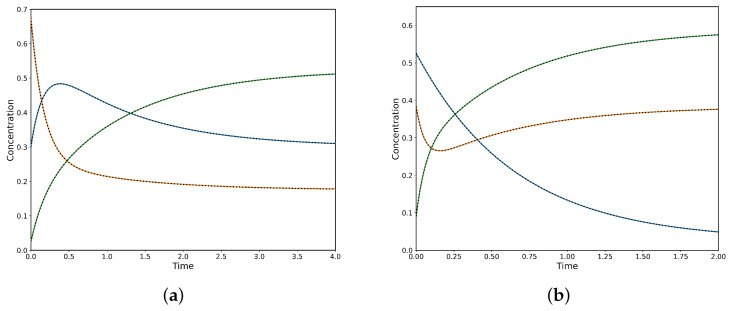
Concentration profiles of species *A*, *B*, and *C* for δ=0.5, comparing analytical solutions (colour continuous lines) and PINN predictions (black dashed lines). (**a**) Case corresponding to a maximum in species *A*, with rate constants $k_{1}^{+}=1.75$, $k_{1}^{-}=3.00$, $k_{2}^{+}=1.50$, and $k_{2}^{-}=0.50\;s^{-1}$. (**b**) Case corresponding to a minimum in species *B*, with $k_{1}^{+}=1.50$, $k_{1}^{-}=0.10$, $k_{2}^{+}=10.0$, and $k_{2}^{-}=6.50\;s^{-1}$.

Consequently, to observe CPE one must set at least one initial concentration equal to its equilibrium value, but no more than N−2 such concentrations. Figure 2 shows two CPE transient regimes (δ=0.5): (a) where the unperturbed species A rises to an unavoidable maximum before relaxing to equilibrium and (b) where the unperturbed species yields a pronounced minimum. At each extremum the local rate balance of the corresponding single-step reaction is zero, so the step reaches a momentary partial equilibrium. The size of the perturbation δ appears as the initial offset from equilibrium for the two perturbed species, while for the linear A→B→C mechanism the extremum timing is independent of δ. Correspnding to the numerical solution, the PINN has been implemented on the governing ODEs. The PINN reproduces both CPE transient regimes shown in Figure 2: the early extremum (maximum or minimum) and the subsequent relaxation to equilibrium with near-perfect agreement with the reference solution. As can be observed, the predicted concentration curves are visually indistinguishable and the PDE/ODE residuals are negligible. The PINN also preserves overall mass balance throughout the transient (with negligible conservation error) and accurately captures the timing and amplitude of the extrema, demonstrating that a data-efficient, physics-constrained model can reliably reproduce CPE dynamics.

Species are classified as either “perturbed” or “unperturbed”. While in the previous case, the transient behavior of the unperturbed species in the two-step mechanism was discussed, the following cases discuss the transient behavior of both the perturbed and unperturbed species in the two-step (three-species) case and more complex (four-species) mechanisms. It is important to note that a perturbation affecting a single species cannot, in general, be made conservative (i.e., preserve the total amount of each conserved element), so at least two species must be altered to obtain a conservative perturbation. Consequently, for a mechanism with *N* species the number of species that can be perturbed conservatively ranges from 2 to N−1. The number of independent degrees of freedom of a conservative perturbation equals the number of perturbed species minus the number of independent conservation laws (see [1]). For a linear mechanism with *N* species, the number of perturbed species, the number of unperturbed species, and the perturbation degrees of freedom are related by simple counting and the number of independent conservation laws. For the three-species case (N=3), a two-species perturbation is considered; hence the number of unperturbed species is 3−2=1. Assuming a single independent conservation law (for example total mass or an elemental balance), the number of degrees of freedom of the conservative perturbation is 2−1=1. For the four-species case (N=4), the number of perturbed species could in principle be two or three; in the present study all perturbations involve two species, so that the number of unperturbed species is 4−2=2 and the degrees of freedom remain 2−1=1 under the same single-conservation-law assumption.

### 4.1. Analysis of Perturbed Species in a Three-Species Acyclic Mechanism

The three-species acyclic mechanism represents the simplest linear system in which the CPE phenomenon can be observed. Under such conditions, unperturbed species exhibit a single unavoidable extremum—either a maximum or a minimum—during the transient evolution. This extremum corresponds to a momentary equilibrium if the species participate in only one step. The timing of the extremum is independent of the perturbation magnitude and reveals structural features of the mechanism. The transient response of perturbed species in two-step mechanisms, i.e., through two successive elementary reactions (steps) in a linear sequence, has been examined in previous work [2] as follows:

$$A\underset{k_{1}^{-}}{\overset{k_{1}^{+}}{⇌}}B\underset{k_{2}^{-}}{\overset{k_{2}^{+}}{⇌}}C.$$

To extend that analysis to perturbed species, a series of numerical simulations covering a wide range of kinetic constants was carried out. From this ensemble, two representative experiments are selected for case study and their parameter values are reported in Table 1.

**Figure 3:**
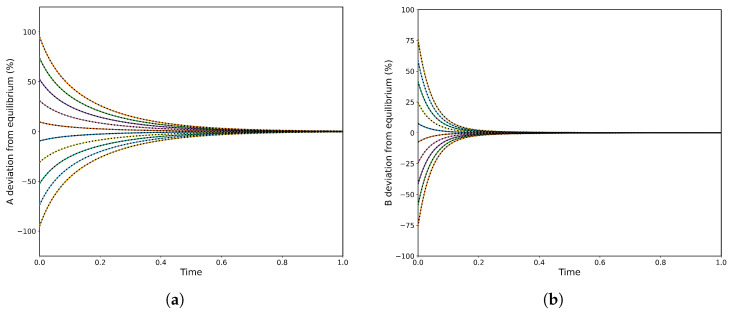

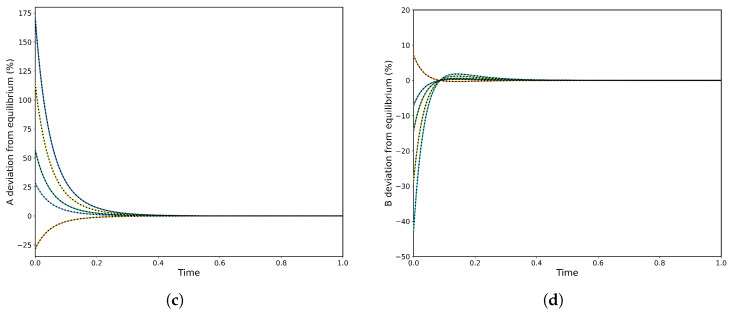
Two-step, three-species acyclic reaction mechanism. Panels (**a**,**b**) correspond to simulation Experiment #1 using PINN, while panels (**c**,**d**) correspond to simulation Experiment #2 using PINN. Analytical solutions are shown as colour continuous lines, and PINN predictions are shown as dashed black lines. (**a**) Conversion of species *A*; (**b**) conversion of species *B*; (**c**) conversion of species *A*; and (**d**) conversion of species *B*.

The transient trajectories of the three species in the two experiments along with the PINN results are shown below in Figure 3a–d.

### 4.2. Analysis of Unperturbed Species in a Three-Species Cyclic Mechanism

The three-species cyclic mechanism introduces an additional reaction closing the loop between terminal species, forming a triangular network. Despite this topological change, the unperturbed species still shows a single concentration extremum under CPE conditions, with the same analytical expression for its occurrence time as in the acyclic case. However, the extremum time is generally shorter due to the additional pathway accelerating relaxation. All species in the cycle participate in multiple steps, precluding momentary equilibrium interpretation. A three-species cyclic mechanism displays an added direct connectivity from species *A* to species *C* as follows: 
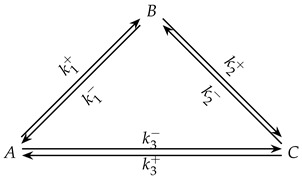


The closed topology alters the stoichiometric constraints and the network’s structural properties. Depending on the elementary step kinetics (mass-action vs. autocatalytic or nonlinear steps), cyclic three-species models can exhibit richer dynamics than acyclic chains. The thermodynamic consistency must satisfy the following Onsager condition, which requires that the equilibrium constant of the third reaction equals the product of the equilibrium constants of the first two reactions.

$$\frac{k_{3}^{+}}{k_{3}^{-}}\;=\;\frac{k_{1}^{+}}{k_{1}^{-}}\;\frac{k_{2}^{+}}{k_{2}^{-}},$$

This enforces detailed balance around the three-reaction cycle and guarantees a single, well-defined equilibrium state. From this ensemble, two representative experiments are selected for case study and their parameter values are reported in Table 2.

**Figure 4:**
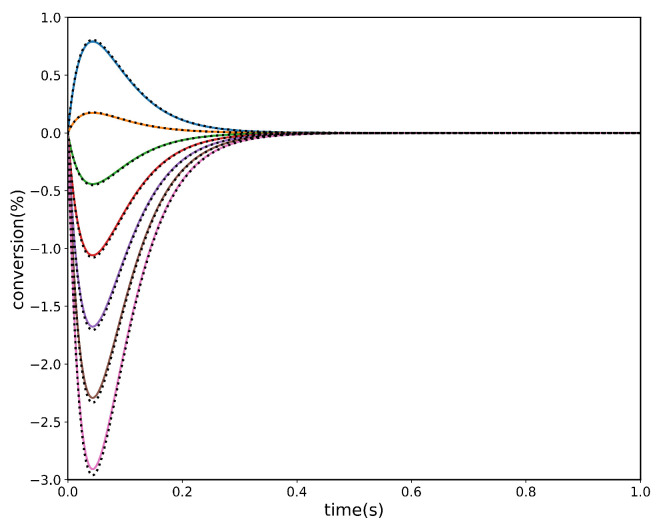
Conversion of the unperturbed species *C* in a two-step, three-species cyclic reaction mechanism. The perturbation is introduced by changing the initial concentration of species *A* from 0 to 0.36 and that of species *B* from 0.92 to 0.56. Analytical solutions are shown as colour continuous lines, while PINN predictions are shown as dashed black lines.

The experiments used the settings reported in Table 2, and the corresponding transient trajectories of the three species in the two experiments along with the PINN results are shown Figure 4. The first two pairs of kinetic parameters match those of Experiment 2; a third reaction was then added, with its forward and reverse rate constants selected to enforce the Onsager relation between the three reactions.

### 4.3. Analysis of Unperturbed Species in a Four-Species Acyclic Mechanism

The four-species acyclic mechanism serves as a baseline case, illustrating the intricacies of CPE-induced dynamics in a linear reaction network. Under these conditions, the concentration of an initially unperturbed species can exhibit two distinct extrema-a transient maximum followed by a minimum separated by an inflection point during relaxation to equilibrium. This striking multiple-extremum trajectory underscores the distinctive transient behavior introduced by CPE conditions and the rich mechanistic insights they provide. The four-species acyclic mechanism is as follows: 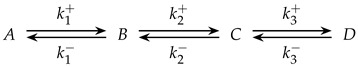
The experimental results of this case are summarized in Table 3.

**Figure 5:**
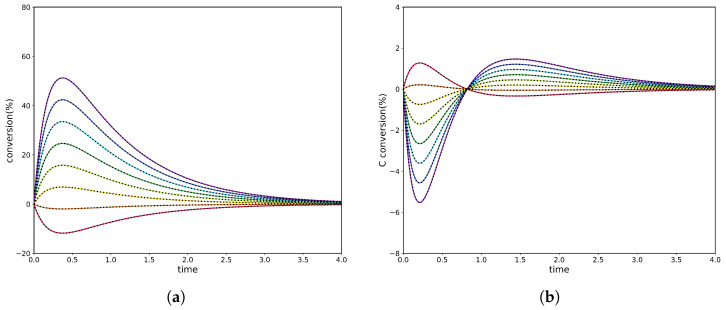
Four-species acyclic mechanism, species *B* and *C* unperturbed: (**a**) conversion of unperturbed species *B* with PINN; (**b**) conversion of unperturbed species *C* with PINN. Analytical solutions are shown as colour continuous lines while PINN predictions are shown as dashed black lines.

**Figure 6:**
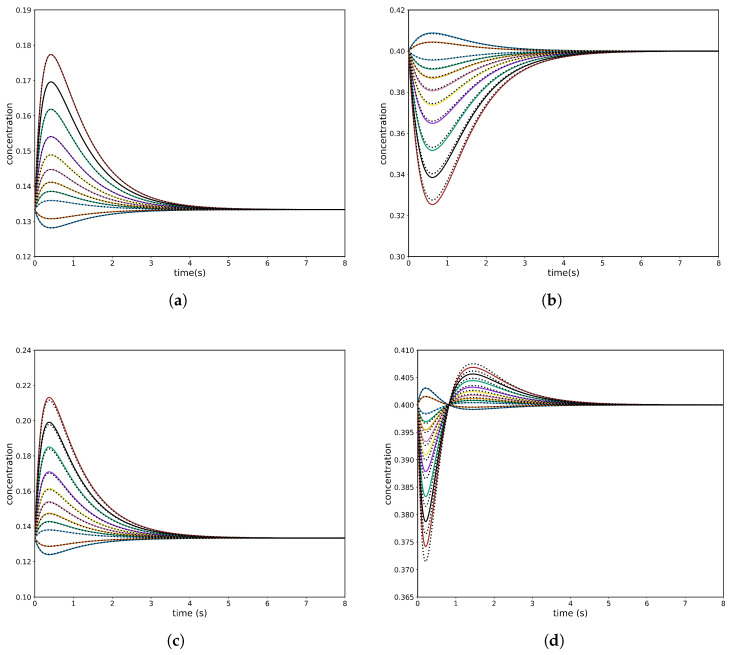
(**a**,**b**) Experiment #2 with PINN: (**a**) concentration of the unperturbed species *B*; (**b**) concentration of the unperturbed species *D*. (**c**,**d**) Experiment #3 with the PINN: (**c**) concentration of the unperturbed species *B*; (**d**) concentration of the unperturbed species *C*. Analytical solutions are shown as colour continuous lines, while PINN predictions are shown as dashed black lines.

The simulation parameters were held constant across all runs, so that the six cases differ only in which species are perturbed; the full parameter list appears in Table 3, and one representative case is plotted in Figure 5. Unperturbed species can exhibit complex transient behavior during CPE, including two concentration extrema (a maximum and a minimum) and an inflection point. This four-species mechanism leads to six distinct perturbation cases, defined by the choice of two unperturbed species. The kinetic parameters and simulation settings for all six combinations are listed in Table 4 and Table 5, while Figure 6 plots two representative cases (Experiment #2 and Experiment #3) that demonstrate this behavior.

### 4.4. Analysis of Unperturbed Species in a Four-Species Cyclic Mechanism

**Figure 7:**
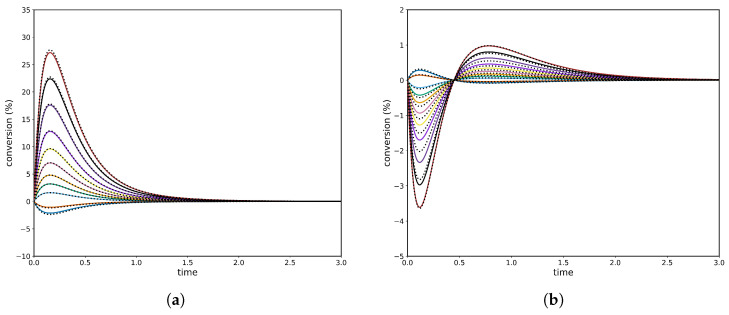
Four-species cyclic mechanism experiment with the PINN, *B* and *C* unperturbed: (**a**) conversion of unperturbed species *B*; (**b**) conversion of unperturbed species *C*. Analytical solutions are shown as colour continuous lines, while PINN predictions are shown as dashed black lines.

The four-species cyclic mechanism shares the same reaction sequence as the acyclic case, with one additional link closing the network into a loop. Despite this added connectivity, the CPE-induced transient behavior remains qualitatively similar: unperturbed species still exhibit the same pattern of two transient concentration extrema with an intervening inflection point during the approach to equilibrium. Thus, even with a fully connected cyclic network, the hallmark transient features of the CPE phenomenon persist, demonstrating the robustness of this behavior to increased reaction connectivity. The four-species cyclic mechanism is as follows: 
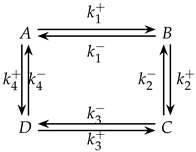


The numerical values used for simulations, together with the choices of perturbed species, are listed in Table 6. Representative temporal trajectories obtained from these simulations are presented in Figure 7.

Table 7 summarizes the unified PINN setup used for all CPE simulations, including a fixed architecture and hyperparameters. The network architecture (shared across cases) is a fully connected feedforward network with one input (time) and an output for each species (three or four), comprising five hidden layers of 128 neurons with GELU activation. Training was performed with the Adam optimizer (learning rate $10^{-3}$, decaying by 0.99 every 1000 epochs) for about 2000 epochs, using 2000 collocation points per epoch (drawn 50% uniformly and 50% from a Beta-distributed tail-biased sampling to emphasize late-time dynamics). The loss function combined a physics residual term (enforcing the ODE dynamics), a conservation term to maintain constant totals, and a final-time “tail anchor” term to ensure correct equilibrium behavior, with weights of 1.0, 0.5, and 2.0, respectively. This core configuration was applied consistently across all cases, and with double-precision training plus validation against a high-accuracy ODE solver (LSODA, tol $10^{-9}$), it enables the PINN to learn the over-equilibrium CPE dynamics accurately while strictly preserving physical constraints.

Table 8 summarizes the system-specific PINN training configurations for each case study, detailing the number of training epochs, collocation points ($N_{f}$), the conservation loss weight ($\lambda_{\mathrm{cons}}$), and any custom adjustments applied in each scenario. Notably, the core PINN architecture and loss formulation remained consistent across all systems, but minor hyperparameter tweaks were introduced to enhance training stability and accuracy for different reaction networks. For example, a higher conservation loss weight ($\lambda_{\mathrm{cons}}=5.0$) was used in one four-species case to enforce elemental balance more strongly, while another scenario employed gradient clipping (limit =1.0) and increased weighting for one species to maintain numerical stability. In addition, a “tail anchor” loss term (with weight 2.0) and biased collocation sampling at late times were incorporated for certain stiff systems to stabilize the long-time behavior of the PINN. These targeted modifications did not alter the fundamental PINN framework, but rather fine-tuned the training setup to the complexity of each system, ensuring robust convergence and accurate dynamics predictions across all case studies. A detailed version of the system-specific PINN training configurations for each case study can be found in Appendix A Table A1. Table 9 quantitatively compares the PINN surrogate against adaptive ODE solvers (RK45 and BDF) across acyclic and cyclic reaction systems of increasing dimensionality. For all cases considered, the PINN achieves $L^{2}$ errors on the order of $10^{-5}$, comparable to high-accuracy adaptive integration, while strictly preserving mass conservation to within $10^{-7}$. Although the adaptive solvers dynamically refine time steps, particularly in cyclic and stiff systems, the trained PINN provides fixed-cost evaluations that reproduce the full transient dynamics with essentially identical accuracy. Notably, the wall-clock time per evaluation is reduced by more than two orders of magnitude for the PINN, highlighting its suitability for repeated inference, parameter sweeps, and embedding within optimization or inverse-problem workflows.

Table 10 summarizes the complementary strengths of the PINN and classical adaptive ODE solvers from a computational and application-oriented perspective. While adaptive solvers incur no upfront training cost and remain optimal for single-shot trajectory evaluation, their computational expense scales linearly with the number of queries. In contrast, the PINN incurs a one-time training cost but delivers sub-millisecond inference thereafter, leading to a break-even point at approximately 10^3^–10^4^ evaluations. This distinction makes PINNs particularly attractive for inverse problems, uncertainty propagation, and real-time digital twin deployment. Importantly, both approaches achieve comparable residual accuracy, confirming that the efficiency gain of the PINN does not compromise physical fidelity.

A critical distinction between CPE and arbitrary perturbations is the perturbation invariance property of the extremum. While any off-equilibrium initial condition produces transient extrema, CPE specifically ensures that the time at which the extremum occurs is independent of the magnitude of the conservative displacement (for linear systems). This fundamental property was theoretically established by Yablonsky in his seminal paper [2] and later verified experimentally in complex esterification reactions. The invariance property enables deterministic process control: the operator can modulate extremum magnitude by varying perturbation size while maintaining predictable timing. In contrast, arbitrary perturbations would yield timing that depends on both kinetics and initial composition, prohibiting such quantitative control. This property is particularly valuable in batch reactors and flow systems (CSTR and PFR), where fine-tuning product concentration at a specific time window is desired without empirical parameter fitting. More recently, Sachs et al. [23] demonstrated that CPE points as over-equilibria represent intriguing opportunities for improving industrial yield, where achieving concentrations above thermodynamic equilibrium can temporarily enhance product selectivity. Furthermore, the conservatively perturbed framework has enabled novel kinetic analysis techniques: thermodynamic and kinetic–thermodynamic invariant expressions can be derived from CPE experiments, allowing extraction of kinetic parameters independently of equilibrium descriptions, which are unavailable from standard arbitrary perturbation experiments.

### 4.5. Application of PINN to Higher-Order Linear CPE Systems

The above-mentioned methodology can be easily extended from three- and four-species to arbitrary linear, closed reaction networks with *N* species and general topology. The state is $c\left(t\right)\in R_{\geq 0}^{N}$ and the dynamics remain linear, as shown in Equation (2). For any reversible link i⇌j with forward rate $k_{ij}^{+}$ and reverse rate $k_{ji}^{-}$, $M_{ij}\leftarrow M_{ij}+k_{ji}^{-}$, $M_{jj}\leftarrow M_{jj}-\left(k_{ji}^{-}\right)$, $M_{ji}\leftarrow M_{ji}+k_{ij}^{+}$, and $M_{ii}\leftarrow M_{ii}-\left(k_{ij}^{+}\right)$, and $M_{pq}$ is zero otherwise. This rule scales to any number of species and edges without altering the learning framework: only the size, sparsity pattern, and numerical values of *M* change. A legal perturbation Δ of the equilibrium must satisfy the linear conservation relations, which are compactly written as LΔ=0 for a fixed $L\in R^{r\times N}$ encoding the *r* independent invariants (e.g., total concentration and elemental balances). The initial state is $c_{0}=c_{eq}+\Delta$, with selected indices left unperturbed by setting $\Delta_{i}=0$ on that subset. The immediate kinematic consequences scale cleanly with network size: because $Mc_{eq}=0$, the initial drift is $\dot{c}\left(0\right)=M\Delta$. Thus, any unperturbed species that is not graph-adjacent to the perturbed set has ${\dot{c}}_{i}\left(0\right)=0$ (“initial shielding”), while species directly adjacent to at least one perturbed neighbor inherit a nonzero initial drift through the corresponding off-diagonal rates. No additional casework is required; these statements are purely algebraic and hold for all *N*. The PINN parameterization also scales without conceptual change. Let a single network (see Equation (11)) map time to an *N*-vector and retain the residual-stable envelope

(17) $$\hat{c}\left(t\right)=c_{0}+g\left(t\right)N_{\theta }\left(t\right),$$

with g(t)=t (or any smooth, strictly increasing function with g(0)=0) and a fully connected subnetwork $N_{\theta }:\left[0,T\right]\to R^{N}$. This guarantees $\hat{c}\left(0\right)=c_{0}$ exactly, regardless of *N*. The time derivative needed for the residual is obtained analytically,

(18) $$\frac{d\hat{c}}{dt}\left(t\right)=g^{\prime }\left(t\right)N_{\theta }\left(t\right)+g\left(t\right)\frac{dN_{\theta }}{dt}\left(t\right),$$

via automatic differentiation. Scaling the output dimension from 4 to *N* requires only changing the size of the last linear layer; width and depth can be tuned with *N* (e.g., H=αN with α∈[1,4] as a practical rule), but the formulation is unchanged. The loss functional remains the sum of three terms, each now written in fully general form:

(19) $$L=\frac{1}{N_{f}}\sum_{i=1}^{N_{f}}∥\frac{d\hat{c}}{dt}\left(t_{i}\right)-M\hat{c}\left(t_{i}\right)∥2^{2}+\frac{\lambda inv}{N_{f}}\sum_{i=1}^{N_{f}}∥L\hat{c}\left(t_{i}\right)-Lc_{0}∥2^{2}+\lambda eq\Phi \left(\hat{c}\left(\cdot \right);c_{eq}\right).$$

The first term enforces the kinetic law at randomly sampled collocation times $\left\{t_{i}\right\}$, the second preserves all invariants in *L* (recovering the familiar $1^{T}\hat{c}\left(t\right)=1$ when $L=1^{T}$), and the third optionally anchors the long-time limit. In higher-dimensional, stiff systems it is often preferable to implement the terminal anchor through a “stationarity” penalty local to late times,

(20) $$\Phi \left(\hat{c}\left(\cdot \right);c_{eq}\right)=\frac{1}{N_{T}}\sum_{j=1}^{N_{T}}∥M\hat{c}\left(T_{j}\right)∥2^{2},\;T_{j}\in \left[T*,T\right],$$

rather than a direct $∥\hat{c}\left(T\right)-c_{eq}{∥}_{2}^{2}$; this avoids biasing trajectories when multiple slow eigenmodes coexist but still drives the prediction into the equilibrium manifold {c:Mc=0}. Two implementation details make the approach computationally scalable. First, evaluation of $M\hat{c}$ exploits sparsity. Because *M* is typically very sparse in large networks, storing it as a compressed sparse matrix and using sparse–dense products reduces the per-batch complexity from $O(N^{2})$ to O(|E|), where |E| is the number of directed edges. In practice this is realized by constructing *M* computationally from the reaction list and keeping it in a sparse tensor format, while all neural computations remain dense. Second, time is non-dimensionalized to temper stiffness: choosing τ=αt with $\alpha \approx |\lambda_{min}\left(M\right)|$ (the largest-magnitude nonzero eigenvalue) contracts the eigenvalue spread toward O(1), which improves gradient flow and reduces the number of required collocation points at early times. Collocation can also be biased toward the boundary layers by sampling τ from a Beta distribution or by the mapping $t=Ts^{p}$ with p>1 to densify near t=0 without altering the loss.

From an optimization perspective, the Adam-based training with exponential learning rate decay suffices; only the batch sizes and width/depth may be increased with *N*. Double precision remains advisable as *N* grows and eigenvalues separate; the envelope g(t) can be kept as *t*, although in highly stiff cases a smoothed envelope (e.g., g(t)=log(1+t) scaled to match *T*) can further stabilize early-time derivatives. Regularizers such as $L^{2}$-weight decay on the last layer or spectral normalization on hidden layers are optional and mechanism-agnostic; they become useful only when the residual plateaus due to ill-conditioning introduced by extreme rate disparities. A detailed form of the main configuration for CPE-PINN systems used in this study, which can be extended to higher-order linear CPE systems, can be found in Appendix A Table A3.

## 5. Conclusions

Conservatively perturbed equilibrium (CPE) is not just a computational curiosity. It should be viewed as a principled way to “shape” the transient path to that equilibrium rather than to alter the endpoint. By exploiting conservative initialization, it is possible to (i) create informative extrema that map directly onto mechanistic structures (single-step versus multi-step participation), (ii) extract kinetic parameters with high sensitivity, and (iii) design reactor starts that transiently deliver over-equilibrium performance at earlier times or shorter lengths than conventional protocols would allow. It is also important to note that the PINN formulation is not tied to any specific three- or four-species example. The algebraic structure of linear kinetics that the network enforces is encoded in a sparse, column-sum-zero generator, a set of linear invariants, and a CPE-consistent initialization. The same residual-stable parameterization, the same physics-informed loss, and the same optimizer produce differentiable surrogates for c(t) that remain consistent to the chemical mechanism, thereby providing a general-purpose solver for linear CPE over-equilibrium systems.

## Figures and Tables

**Table 1:** Two-step, three-species acyclic conservatively perturbed equilibrium (CPE) example with settings.

Experiment Settings	Experiment #1	Experiment #2
Kinetic Parameters (s^−1^):	$k_{1}^{+}=5,\;k_{1}^{-}=4$ $k_{2}^{+}=12,\;k_{2}^{-}=6$	$k_{1}^{+}=16,\;k_{1}^{-}=4$ $k_{2}^{+}=12,\;k_{2}^{-}=6$
Perturbed species:	*A*, *B*	*A*, *B*
Unperturbed species:	*C*	*C*

**Table 2:** Three-species cyclic conservatively perturbed equilibrium (CPE) example with settings.

Experiment Settings	Value
Kinetic parameters (s^−1^):	$\begin{array}{cccc} k_{1}^{+} & =16\; & k_{1}^{-} & =4\\ k_{2}^{+} & =12\; & k_{2}^{-} & =6\\ k_{3}^{+} & =8\; & k_{3}^{-} & =1 \end{array}.$
Perturbed species:	*A*, *B*
Unperturbed species:	*C*

**Table 3:** Four-species acyclic conservatively perturbed equilibrium (CPE) example with settings.

Experimental Settings	Value
Kinetic parameters ($s^{-1}$):	$k_{1}^{+}=2$ $k_{1}^{-}=1$ $k_{2}^{+}=3$ $k_{2}^{-}=1$ $k_{3}^{+}=1$ $k_{3}^{-}=1$
Perturbed species:	*A*, *D*
Unperturbed species:	*B*, *C*

**Table 4:** Four-species acyclic conservatively perturbed equilibrium (CPE) example with experimental settings.

Experimental Settings	Values
Kinetic parameters (s^−1^):	$k_{1}^{+}=2$ $k_{1}^{-}=1$ $k_{2}^{+}=3$ $k_{2}^{-}=1$ $k_{3}^{+}=1$ $k_{3}^{-}=1$

**Table 5:** Four-species acyclic conservatively perturbed equilibrium (CPE) example with cases and results.

Experiment	Perturbed Species	Unperturbed Species	Behavior
1	*A*, *B*	*C*, *D*	2 extrema of [*C*], 1 of [*D*]
2	*A*, *C*	*B*, *D*	1 extremum of [*B*], 1 of [*D*]
3	*A*, *D*	*B*, *C*	2 extrema of [*C*], 1 of [*B*]
4	*B*, *C*	*A*, *D*	1 extremum of [*A*], 1 of [*D*]
5	*B*, *D*	*A*, *C*	1 extremum of [*A*], 1 of [*C*]
6	*C*, *D*	*A*, *B*	1 extremum of [*A*], 1 of [*B*]

**Table 6:** Four-species cyclic conservatively perturbed equilibrium (CPE) example with settings.

Experiment Settings	Value
Kinetic parameters (s^−1^):	$k_{1}^{+}=2$ $k_{1}^{-}=1$ $k_{2}^{+}=3$ $k_{2}^{-}=1$ $k_{3}^{+}=1$ $k_{3}^{-}=1$ $k_{4}^{+}=1$ $k_{4}^{-}=6$
Perturbed species:	*A*, *D*
Unperturbed species:	*B*, *C*

**Table 7:** Concise form of the main configuration for CPE-PINN systems used in this study.

Category	Parameter	Value
**Network **	Architecture	1→128→128→128→128→128→3/4
	Activation	GELU
	Parameterization	$c\left(t\right)=c_{0}+t\cdot N\left(t\right)$
**Training**	Optimizer	Adam ($lr=10^{-3}$)
	Scheduler	ExponentialLR (γ=0.99, every 1k epochs)
	Epochs	2000 (standard) / 2500
	Collocation Points ($N_{f}$)	2000–2200 per epoch
**Sampling**	Strategy	50% uniform + 50% Beta(3,1) tail-biased
**Loss**	Physics ($\lambda_{\mathrm{phy}}$)	$1.0\times \mathrm{MSE}({\frac{dc}{dt}}_{\mathrm{PINN}},{\frac{dc}{dt}}_{\mathrm{ODE}})$
	Conservation ($\lambda_{\mathrm{cons}}$)	$0.5\times \mathrm{MSE}(\sum c_{i},1)$
	Tail Anchor ($\lambda_{\mathrm{tail}}$)	2.0×MSE(residual@t=T) [256 pts]
**Precision**	dtype	torch.float64
**Validation**	Method	solve_ivp (LSODA, tol = $10^{-9}$)

**Table 8:** Concise system-specific configuration for CPE-PINN systems used in this study.

Figure	System	Epochs	$N_{f}$	$\lambda_{\mathrm{cons}}$	Modifications
Figure 3a–c	3-sp cyclic	800	1000	0.5	Standard
Figure 3d	3-sp cyclic	1000	1000	0.5	Standard
Figure 4	3-sp cyclic	2000	1200	0.5	No tail anchor
Figure 5a	4-sp acyclic	2000	2000	5.0	High conservation weight
Figure 5b and Figure 6a–c	4-sp acyclic/cyclic	2000	2000	0.5	Standard
Figure 6d	4-sp acyclic	2500	2200	0.5	+Grad clip (1.0), 1.6× weight for C
Figure 7a,b	4-sp cyclic	2000	2000	0.5	+Tail anchor (2.0), Tail-biased collocation markers

**Table 9:** Comparison of PINN and adaptive ODE solvers (RK45 and BDF) for acyclic and cyclic reaction systems with increasing species complexity.

Metric	PINN	ODE (RK45, Adaptive)	ODE (BDF, Adaptive)	Notes
**3-Species Acyclic System**				
$L_{2}^{2}$ Error	$1.2\times 10^{-5}$	$1.4\times 10^{-5}$	$1.3\times 10^{-5}$	Comparable accuracy
Mass conservation error	<$10^{-7}$	<$10^{-7}$	<$10^{-7}$	All satisfy physical constraints
Adaptive steps taken	2000 (fixed)	127	145	RK45 refines during transient
Wall-clock time (single eval)	<1 ms	95 ms	112 ms	ODE solver slower for single-shot inference
**3-Species Cyclic System**				
$L_{2}^{2}$ Error	$1.1\times 10^{-5}$	$1.5\times 10^{-5}$	$1.2\times 10^{-5}$	Comparable accuracy
Mass conservation error	<$10^{-7}$	<$10^{-7}$	<$10^{-7}$	All satisfy conservation
Adaptive steps taken	2000 (fixed)	156	168	Higher stiffness detected
Wall-clock time (single eval)	<1 ms	115 ms	128 ms	ODE slower; BDF handles stiffness
**4-Species Acyclic System**				
$L_{2}^{2}$ Error	$1.4\times 10^{-5}$	$1.6\times 10^{-5}$	$1.5\times 10^{-5}$	Comparable; slight BDF advantage
Mass conservation error	<$10^{-7}$	<$10^{-7}$	<$10^{-7}$	All maintain physical validity
Adaptive steps taken	2000 (fixed)	203	218	Increased complexity; more steps
Wall-clock time (single eval)	<1 ms	142 ms	155 ms	ODE cost increases with system size
**4-Species Cyclic System**				
$L_{2}^{2}$ Error	$1.5\times 10^{-5}$	$1.7\times 10^{-5}$	$1.4\times 10^{-5}$	BDF superior in cyclic/stiff case
Mass conservation error	<$10^{-7}$	<$10^{-7}$	<$10^{-7}$	All conserve mass effectively
Adaptive steps taken	2000 (fixed)	187	211	Highest stiffness; BDF more efficient
Wall-clock time (single eval)	<1 ms	138 ms	149 ms	Comparable to RK45 despite more steps

**Table 10:** Comparison of PINN and adaptive ODE solvers in terms of computational cost, accuracy, and application suitability.

Metric	PINN	ODE Solver (Adaptive)	Advantage
Training time	5–10 min	0	ODE
Single evaluation	<1 ms	50–200 ms	PINN (50–200×)
Break-even queries	≈4000	–	PINN
Residual accuracy	$10^{-5}$–$10^{-6}$	$10^{-5}$–$10^{-6}$	None
Storage size	<5 MB	Model code	PINN
Inference accuracy	Marginal extrapolation loss	Stable and bounded	ODE
Application fit	Inverse problems, Monte Carlo sampling	Single-shot evaluation	Domain-dependent

## Data Availability

No new data were created or analyzed in this study. Data sharing is not applicable to this article.

## Appendix A

### Appendix A.1

Table A1 serves as a comprehensive reference for the specific hyperparameters and architectural configurations of the physics-informed neural network (PINN) used to generate the results in Figure 3, Figure 4, Figure 5, Figure 6 and Figure 7. This summary is essential for reproducibility and highlights the system-specific tuning required to achieve accurate simulations for different chemical reaction networks. The table documents the progression of this study, beginning with three-species cyclic systems (Figure 3 and Figure 4) and advancing to more complex four-species acyclic (Figure 5 and Figure 6) and cyclic (Figure 7) mechanisms. For each case, it specifies the model’s training duration (Epochs) and the number of collocation points ($N_{f}$) used to enforce the physical constraints. Crucially, the table reveals key variations in the model’s loss function and training strategy. Early experiments (Figure 3 and Figure 4) omitted the tail anchor loss, a feature introduced in later models (Figure 5 and Figure 6) to improve the solution’s stability and accuracy at the final time ($t=T_{END}$). Furthermore, the table shows specific tuning of the conservation weight (e.g., increased to 5.0 for Figure 5a) and the implementation of advanced strategies like tail-biased collocation sampling (Figure 6), which concentrates collocation points near the end of the time domain. This fine-tuning, including species-specific weighting of the physics loss (e.g., “1.6× for C” in Figure 6d), underscores the necessity of adapting the PINN architecture and loss formulation to the unique dynamic characteristics of each CPE experiment.

**Table A1:** Detailed form of the system-specific variations for CPE-PINN systems used in this study.

Figure	System Type	Species	Epochs	$N_{f}$	Cons. Weight	Special Features
Figure 3a–c	3-species cyclic	*A*, *B*, *C*	800	1000	0.5	No tail anchor used
Figure 3d	3-species cyclic	*A*, *B*, *C*	1000	1000	0.5	No tail anchor used
Figure 4	3-species cyclic	*A*, *B*, *C*	2000	1200	0.5	No tail anchor used
Figure 5a	4-species acyclic	*A*, *B*, *C*, *D*	2000	2000	5.0	Higher conservation penalty; asymmetric δ
Figure 5b	4-species acyclic	*A*, *B*, *C*, *D*	2000	2000	0.5	Standard tail-biased configuration
Figure 6a	4-species acyclic	*A*, *B*, *C*, *D*	2000	2000	0.5	Tail-biased collocation
Figure 6b	4-species acyclic	*A*, *B*, *C*, *D*	2000	2000	0.5	Tail-biased collocation
Figure 6c	4-species acyclic	*A*, *B*, *C*, *D*	2000	2000	0.5	Tail-biased collocation
Figure 6d	4-species acyclic	*A*, *B*, *C*, *D*	2500	2200	0.5 (1.6× for *C*)	Gradient clipping; species-weighted physics
Figure 7a,b	4-species cyclic	*A*, *B*, *C*, *D*	2000	2000	0.5	Markers every 60 points for visualization

### Appendix A.2

The physics-informed neural network (PINN) used to model the conservatively perturbed equilibrium (CPE) dynamics is structured as a five-layer feedforward MLP with 128 neurons per layer and a GELU activation function. Training is performed using the Adam optimizer with an initial learning rate of $1\times 10^{-3}$ and an exponential decay scheduler. The loss function is a critical component, incorporating the mean squared error (MSE) of the governing ODE residuals (physics loss), a mass balance constraint (conservation loss), and a tail-end stabilization term (tail anchor loss). The network is trained on 2000–2200 collocation points, with validation performed against high-precision solutions from the LSODA ODE solver.

**Table A2:** PINN architecture, training configuration, and numerical settings used in this study.

Component	Setting	Notes
Network	5-layer MLP, 128 neurons/layer	GELU activation, linear output
Input/Output	1 input (*t*)/3 or 4 outputs (species)	Matches problem dimensionality
Parameterization	$c\left(t\right)=c_{0}+t\cdot N\left(t\right)$	Satisfies initial condition by construction
Optimizer	Adam, $\mathrm{lr}=10^{-3}$	Exponential decay γ=0.99 every 1000 epochs
Epochs	2000–2500	Longer for stiff or cyclic cases
Collocation Points	2000–2200 per epoch	50% uniform, 50% tail-biased (Beta(3,1))
Loss Terms	Physics (1.0), Conservation (0.5–5.0), Tail Anchor (2.0)	Adjustable per case
Precision	Double (float64)	Ensures stability and accuracy
Validation	LSODA (SciPy) with tol = $10^{-9}$	High-precision reference integration
Gradient Handling	Clipping (1.0) in some cases	Used when numerical instability arises
Device	CUDA if available	Defaults to CPU otherwise

**Table A3:** Detailed main configuration for CPE-PINN systems.

PINN Component	Configuration	Notes
**Architecture**		
Network Type	Feedforward MLP	Sequential MLP
Input Dimension	1	Time (*t*)
Output Dimension	3 or 4	Species conc. (*A*, *B*, *C*) or (*A*, *B*, *C*, *D*)
Hidden Layers	5	Excl. input and output layers
Neurons per Layer	128	Uniform across hidden layers
Activation Function	GELU	Gaussian Error Linear Unit
Output Activation	None (linear)	Direct concentration output
**Parameterization**		
Concentration Form	$c\left(t\right)=c_{0}+t\cdot N\left(t\right)$	Initial condition satisfied automatically
N(t)	Neural network output	Learned time-dependent correction
**Training**		
Optimizer	Adam	Adaptive moment estimation
Learning Rate (lr)	$1\times 10^{-3}$	Initial lr
LR Scheduler	ExponentialLR	Multiplicative decay
Scheduler Gamma (γ)	0.99	Applied every 1000 epochs (typ.)
Epochs	2000–2500	2000 standard; 2500 for Figure 6d
Batch Size ($N_{f}$)	2000–2200	Collocation pts/epoch
**Collocation Strategy**		
Base Sampling	Uniform random	50% points (60% in Figure 6d)
Tail-Biased Sampling	Beta(3,1)	Dense near $t=T_{\mathrm{END}}$
Endpoint Inclusion	Optional	Include t=0 and/or t=T
**Loss Function**		
Physics Loss	$\mathrm{MSE}\;\left({\dot{c}}_{\mathrm{PINN}},{\dot{c}}_{\mathrm{phys}}\right)$	ODE residual
Conservation Loss	$\mathrm{MSE}\;\left(\sum_{i}c_{i},1.0\right)$	Mass balance constraint
Tail Anchor Loss	MSE at $t=T_{\mathrm{END}}$	Stabilizes late-time behavior
Physics Weight	1.0	Base weight
Conservation Weight	0.5–5.0	0.5 standard; 5.0 for Figure 5a; 1.6× for *C* in Figure 6d
Tail Anchor Weight	2.0	Applied to 256 points at $t=T_{\mathrm{END}}$
**Gradient Management**		
Gradient Clipping	1.0 (norm)	Applied in Figure 6d only
Autograd Method	torch.autograd.grad	Per-species derivative computation
Gradient Graph	create_graph=True	For higher-order derivatives
retain_graph	True	Keep graph for multiple species
**Numerical Precision**		
Data Type	torch.float64	Double precision
Device	CUDA (if available)	Falls back to CPU
**Time Domain**		
$t_{\mathrm{span}}$	System-dependent	(0, 1–8) s (typ.)
Evaluation Points	600–1200	For plotting and validation
**Validation**		
Reference Method	scipy.integrate.solve_ivp	LSODA method
ODE Tolerances	rtol = 1 × 10^−9^, atol = 1 × 10^−9^	High-precision validation
